# What motivates physicians to propose private services in a mixed private-public healthcare system? A mixed methods study

**DOI:** 10.1186/s12913-022-07474-9

**Published:** 2022-01-10

**Authors:** Tal Michael, Dani Filc, Nadav Davidovitch

**Affiliations:** 1 School of Public Health, Ben-Gurion University of the Negev, POB 653, 84105 Be’er-Sheva, Israel; 2 Department of Politics and Government, Ben-Gurion University of the Negev, Be’er- Sheva, Israel

**Keywords:** Private public mix, Privatization, Healthcare, Private insurance, Mixed methods

## Abstract

**Background:**

Implementation of private elements, including private insurances, in public healthcare system is now common in many countries, and its impacts have been well studied. Little, however, is known about the motives leading physicians, major role players in the system, to promote the usage of private services. The aim of this study was to explore the various motives leading physicians within public systems to propose private services to their patients, while examining the possible associations to their specialty and level of commitment.

**Methods:**

A total of 197 physicians from specialisms loaded more to private/public sectors participated in a cross-sectional telephone survey regarding their attitudes on their practices, private insurances, access to healthcare, and job satisfaction. The association between the likert scale questions to their recommendation to purchase private insurance, and the commitment they felt towards patients were analyzed using Generalized Estimating Equations (GEE) as well as logistic regression models.

**Results:**

Our findings suggest physicians engaged in dual practice are less likely to promote private insurances among their patients if they are satisfied with their public job (OR = 0.92, 95%CI 0.89,0.94). Physicians perceived private insurances as beneficial for patients, were found likely to promote them (OR = 1.65, %95CI 1.16, 2.35). The commitment physicians felt toward patients who paid out-of-pocket money was associated to their sense of being trusted and valued (OR = 1.99, 95%CI 1.33, 2.88; OR = 1.5, 95%CI 1.05, 2.13 respectively).

**Conclusion:**

This study suggests a deeper understanding of physicians’ daily experience of the private-public mix and it’s consequences, and could provide a platform for future studies. Further studies on physician’s role in health privatization processes are needed, and could aid policymakers in their efforts to strengthen healthcare systems around the world.

**Supplementary Information:**

The online version contains supplementary material available at 10.1186/s12913-022-07474-9.

## Background

Many healthcare systems around the world have adopted a private-public mix (PPM) approach in recent years [1] as a way to cope with growing healthcare expenses under increasing budget constraints [2]. While there are several models of PPM, the common principle is sharing the risks and rewards between public and private stakeholders [3]. Those who endorse these models believe that private-public collaboration can reduce public healthcare expenditure [1] and, if well regulated, save large sums of money by focusing on patient outcomes, leveraging more from public assets, and improving service quality and diversity [3]. Alongside the potential advantages of PPM, there are some issues that cause major concern, such as the potential for the complexity of the combined system leading to inequities, higher out-of-pocket expenditure, and inefficient distribution of resources [4]. Opponents of PPM also claim that imposing private-public collaboration on healthcare systems can lead to conflicts of interest, as services provided by private organizations are not always compatible with the values of the public system [5].

PPM comes in many forms, and choosing how to integrate private elements into the public sector system depends both on the role of public regulators and the degree of risk and responsibility the private sector is willing to take. A 1991 World Health Organization (WHO) report points out that the terms “private sector” and “privatization” can apply either to the financing or provision of health-related services [6]. Barlow et al. [1] propose a typology for PPM models according to the different combinations of finance and service provision. In the United Kingdom, for example, the Private Finance Initiative (PFI) was the principal model of PPM, with the National Health Service (NHS) still responsible for providing clinical services [6] but with capital investments supplied by the private sector [7]. Once considered a breakthrough in health supply [8], the PFI these days perceives by many experts as a failure and slowly extinguishes [9] as the British government seeks for solutions with higher value-for-money. In Germany, the public authorities may transfer public hospitals to private franchises. In the public financing/private provision model, currently one of the world’s most common models, some services are supplied by the private sector based on the assumption that their quality and accessibility are better than they would be if provided by the public sector and, if well-regulated, even enhance value for money [10]. In Australia, the private sector is integrated with the public health services. All citizens are covered by the national health program, but services received outside of the major public hospitals – such as GP appointments and certain diagnostic tests [11] – are provided by private practitioners on a fee-for-service basis, with citizens reimbursed to a level defined by law [12]. In other countries, PPM features in the expansion of private health insurance (PHI) as a way to finance new services [12]. Although PHI adds to consumer choice and makes institutions more responsive, among other benefits, it also increases both individual and collective health expenditure while exacerbating sociodemographic as well as age-related inequities [13, 14]. Shaoul [15] claimed the benefits of PPM were concentrated among those who could afford purchasing PHI, while the costs were diffused throughout the population, increasing the polarization and deepening the inequalities. Furthermore, it was found associated to lower accessibility to health services among patients in the public sector [14], and eventually associated to cost inflations and decrease in the value of money paid by individuals and collectives [16]. Comprehensive financial analysis [15] found that penetration of market forces into health systems was dangerous; driven by “efficiency gain”, the private system willing to maximize patient flow, and could easily harm the treatment of chronically ill patients who considered unprofitable. According to Shaoul, eventually the costs of treatment could pass onto the public as well as the social services systems and patients families.

Many studies have examined the implementation of PPM in healthcare systems around the world [4, 7, 17] and the effects it has had on access to treatment, on patients, and on systems in general. Few, however, have focused on the major role physicians play in these partnerships and the influence they have over the form of their development [18–21]. As frontline staff, physicians interact with patients and implement health regulations. Moreover, they play a crucial role in patient decision-making processes over key matters such as courses and providers of treatment [18]. PPM places physicians at the interface of public and private sectors in healthcare, and can therefore play a major role in the implementation of the various forms of PPM. Most studies of physicians’ perceptions in this regard have examined their motivations to engage in dual public- and private-sector practice [19, 22, 23] rather than identifying what motivates physicians to promote the services they provide to their patients within private frameworks .

Under Israel’s National Health Insurance Law, the country’s health funds provide universal healthcare services that are financed by the state according to a standardized capitation system [24]. As in other OECD countries, the financing of healthcare in Israel relies on a mix of public and private sources, but the share of private financing in Israel has lately reached a peak of 37%, significantly higher than the 26% OECD average [25]. The main form PPM takes in Israel is the provision of PHI by the public health funds a combination of supplemental and duplicate insurance.

Though PHI provides significant healthcare financing in many OECD countries to various degrees, it is particularly prevalent in Israel. A 2017 survey found that nearly 84% of respondents said they subscribed to at least one PHI scheme, and more than one-third that they subscribed to more than one [13]. Those who have PHI have the privilege of visiting specialists for the same problem in both the public and private sectors, increasing “doctor shopping’ [26]. It has also boosted supplemental and duplicate insurance and covers a number of diagnostic procedures, drug provisions, and second opinions. It furthermore allows patients to choose their hospital surgeons in both private hospitals and certain, mainly Jerusalem-based, public ones. It is the most salient example of the blurring of boundaries between the Israeli private and public sectors, since PHI is sold by the health funds themselves. Each health fund may include its own set of supplemental services to its plans, as long as that it does not include lifesaving procedures and treatments, and as long as there is no underwriting. Over time, health funds have increased the number and range of private services in the PHIs they sell. In another manifestation of PPM’s reach, the two biggest health funds own private hospitals which provide services to patients owning private insurance sold by those public health funds from physicians who also work in the public system [27].

Physicians may choose to work only in public healthcare, only in private healthcare, or in both (dual practice) [28]. The increasing demand for private healthcare resulting from the expansion of PHI affects the work dual practice physicians undertake. Up until 2017, when new regulations were implemented, it was easy for those in dual practice to transfer their patients, with patient consent, from the public system to their private, fee-paying clinics, where the waiting times are shorter, charging them for each procedure and appointment if both agreed. The drivers of physician’s choice to increase private patients could be associated to debt or family size [29] and to wage incentives [30], though the effect of the last varied among different studies [21, 31], and requires further understanding. The widely adopted private practice exacerbated social inequalities, since research has shown that it increases waiting times for procedures in the public system, adversely affecting those who are not able to purchase PHI [32].

It is important to assess the role physicians play in the process in order to understand why such a very high percentage of people purchase PHI, coverage which mostly duplicates existing insurance or covers non-essential, “nice-to-have” services. Our study analyzes physicians’ perceptions of and attitudes to PHI and whether and to what extent they play a role in convincing patients to subscribe to it. Given the major changes in the healthcare system in recent years, it has become crucial to perform this examination.

In order to make such an assessment, a prior qualitative research was conducted among 21 physicians in both the private and public sectors between November 2016 and August 2017 [33]. These included specialists from residencies where private practice was conducted for less frequently accessed areas of treatment in infectious disease and intensive care, and for the more commonly used services of orthopedics and cardiology. We chose these specialties following a prior descriptive analysis that showed that the first two were strongly associated with private healthcare, while the second two were more strongly associated with public sector healthcare. In-depth, semi-structured interviews were used to understand the participants’ views on PHI. This analysis and its results have been described in detail in a previous paper [33]. Thematic coding analysis of the interviews identified certain themes: privatization; an increase in PHI usage; and the relationships between physicians and patients [34, 35]. These themes were categorized at three levels according to the perceptions of the physicians of privatization in general and PHI particularly: the macro-, micro-, and practice levels. As part of an integrated methodology, the qualitative research findings served as the basis for the quantitative survey that is the focus of this article.

## Objectives

The overall objective of this study was to examine the various motives leading physicians to propose private services to their patients in a mixed private-public healthcare system.

Using the motives elicited in the qualitative interviews, the key aim was understanding the reasons leading physicians to suggest their patients to purchase or use existing PHI. The secondary aims included examining the possible association between the nature of the physicians’ residency (private or public) to their suggestions and understanding what affects physicians’ commitment to patients who pay.

## Methods

### Study design and participants

The qualitative component of the research was followed up with a standardized survey (via telephone), conducted between August 2018 and January 2019, of physicians from all four major health funds from both the public and private sectors, randomly chosen from lists supplied by the Israeli Medical Association (IMA). Interviews were conducted in Hebrew by the Tel Aviv-based Geocartography Knowledge Group company. The criteria for inclusion were that participants were: (1) members of the IMA; (2) currently working physicians; (3) working in at least one framework; (4) working in one of the four major health funds mentioned above; (5) Hebrew-speaking Israeli citizens. We excluded physicians who did not study medicine in Israel, in order to neutralize the potential effects their medical education had on their outlook [36] and also those over 70 years of age. Out of 300 cardiologists and orthopedic surgeons, 160 randomly chosen agreed to participate (53%) and, of those, 14 were excluded for the above reasons of age or foreign education. The survey elicited responses from 197 physicians: 51 from the public sector and 146 from the private or private-public dual sectors. The physicians chosen were specialists from four different residencies in the following proportions: cardiology (77) orthopedic surgery (66), critical care (23) and infectious diseases (28). Among the 50 critical care physicians and 42 infectious diseases physicians approached, 46 and 66% respectively agreed to participate.

### Data collection

The qualitative data analysis presented previously [33] elicited 70 codes which were categorized into 12 themes. Based on the themes and motives identified in the previous study, surveys were drafted and pilot testing of the reworded questionnaire conducted on 30 physicians. The internal consistency of the various subjects tested was then tested, using Cronbach’s alpha coefficient [37]. Analysis of each subject was performed, and questions that decreased the coefficient (< 0.7) of each subject were excluded, as accepted in the literature [38]. The final survey consisted of 40 questions in six sections: on background characteristics (five open-ended questions); on demographics and practice (five closed-ended questions); on perceptions on access to healthcare (five Likert scale questions); attitude toward private insurances (three Likert scale, and four closed-ended questions); on job satisfaction (seven Likert scale questions); and on work satisfaction in the public sector (nine Likert scale questions). We used four Likert scale-style questions in order to avoid neutral answers [39], while also allowing participants to declare the question “irrelevant.” For each statement, participants had to choose one of five responses: “Strongly disagree,” “Disagree,” “Agree,” “Strongly agree,” or “Irrelevant.” The protocols for both stages were approved by the Institutional Ethics and Human Subjects Review Committee of Ben-Gurion University of the Negev.

### Variables and measurements

The dependent binary variables were the answers for two “yes/no” questions regarding each of our outcomes: (1) “Will you recommend your patient using/purchasing PHI?”; (2) “Do you feel more obliged to patients who pay out-of-pocket for the service they receive?” The questions addressed doubts and issues arising from the previous stage of interviews conducted with physicians [33]. The covariates included demographic details: gender, age, and place of residence. It also included professional data: the institution from which they graduated; the district they work in; the framework within which they operate; the nature of their residency; their years of seniority as a specialist; approximate number of patients seen in the private health system per week; and the approximate number in the public health system per week. The other independent variables related to the various questionnaire topics: perceptions of access to healthcare; attitude toward PHI; job satisfaction; and the satisfaction of work in the public sector.

### Statistical analysis

Univariate analysis was performed using *χ*^2^ and Fisher Exact tests between the nominal variables and the outcome variables. The relation between the dichotomous and continuous numerical variables that were normally distributed were examined using Student’s t-tests. The relations between dichotomous variables and non-continuous numerical variables, including the ordinal questionnaire answers, were examined using non-parametric Mann Whitney tests.

A multivariate Generalized Estimating Equations (GEE) regression was then performed in order to assess the association between the binary outcomes and the covariates, using the different residency types (public/private) as clusters. Multivariate logistic regression analyses were performed when only one population was analyzed. Variables found significant (*p* < 0.1) in the univariate tests, were gradually added to the regression after examination of possible confounders and the interaction among the independent variables. Final GEE regression modeling was performed using an unstructured matrix, as it was found most appropriate for each one of them using Quasi-likelihood under Independence Model Criterion (QIC). Models were compared using corrected QIC (QICC) as common in the literature [40]. Logistic models on the other hand were compared using -2LL and Receiver Operating Characteristic (ROC) analysis.

## Findings

### Physicians population characteristics

Table 1 characterizes the physicians surveyed by the orientation of their residency. Most were found to be males (83.8%, *n* = 165) and most engaged in the public and private sectors (71.9%, *n* = 120). Of the females, only eighteen (54.5%) engaged in such dual practice. There were no significant differences in the average age of both genders (U = 3283, *P*-value = 0.813). No significant differences were found among physicians from both sectors with regard to time working in their profession and time in both residency and fellowship (U = 3191, *P*-value = 0.927; U = 2925,*P*-value = 0.878 respectively). The majority worked in the northern (57.1%, *n* = 109) and central (30.9%, *n* = 59) districts of Israel, and there was no significant difference between the two sectors regarding the working districts (*χ*^2^ = 1.699, *P*-value = 0.428). Physicians oriented toward private practice saw a significantly higher number of patients per week on average (72.61 ± 23.15 vs 48.00 ± 23.00, U = 2000, *P*-value < 0.001), and their median number of frameworks was significantly higher too (3.0 compared to 1.0, U = 1993, P-value < 0.001). Most engaged in dual practice (78.1%, *n* = 114), while fewer of the physicians oriented towards the public sector engaged in dual practice (41.2%, *n* = 21).

**Table 1:** Background characteristics of respondents by their residency: private- versus public-sector loaded specialism

	Private loaded specialism	Public loaded specialism	*P*-value	*U*/*χ*^2^
	*n = 146*	*n = 51*		
*Age* [years] (Mean ± S.D)	57.95 ± 9.3	57.64 ± 8.86	0.813	3283
*Gender*				
Males n (%)	132 (90.4)	33 (64.7)	< 0.001	18.357
Females n (%)	14 (9.6)	18 (35.3)		
*Residency and fellowship details*				
Years since end of residency (Mean ± S.D.)	21.63 ± 10.18	22.0 ± 10.56	0.927	3191
Duration of residency and fellowship (years) (Mean ± S.D.)	7.03 ± 1.71	7.07 ± 1.30	0.878	2925
*Working district*				
North n (%)	85 (59)	25 (51.1)	0.428	1.699
Center n (%)	44 (30.6)	15 (31.9)		
South n (%)	15 (10.4)	8 (17)		
*Working frameworks*				
Working in public hospital n (%)	110 (75.3)	47 (92.2)	0.010	6.604
Working in private hospital n (%)	45 (30.8)	5 (9.8)	0.003	8.816
Employee in health fund n (%)	31 (21.2)	6 (11.8)	0.136	2.221
Semi-private clinic n (%)	45 (30.8)	8 (15.7)	0.036	4.403
Private clinic n (%)	41 (28.1)	4 (7.8)	0.003	8.784
Number of frameworks (Median)	3	1	< 0.001	1993
Engaging in dual practice n (%)	114 (78.1)	21 (41.2)	< 0.001	23.87
Estimated number of patients [per week] (Mean ± S.D)	72.61 ± 23.15	48.0 ± 23.0	< 0.001	2000

### Physicians’ motivations in promoting private services to their patients

In the univariate analysis, we examined the association between the dichotomous response for the question “Will you recommend your patient using or purchasing a private health insurance?” as a dependent variable, and the background characteristics, as well as other questions (Table 2). Contrary to our hypothesis, the recommendation to purchase PHI was not significantly associated with most of the background characteristics, including the physician’s specialty (*p*-value = 0.994).

**Table 2:** Characteristics of physicians responded to the statement: “Will you recommend using or purchasing a PHI to your patient?”

	Will you recommend PHI to your patient?”		Odds Ratio	*P*-value	CI95% - Low	CI95% - High
	Yes	No				
	*n* = 115	*n* = 68				
*Gender*						
Males n (%)	98 (83.1)	57 (83.8)	0.946	0.892	0.423	2.115
Females n (%)	20 (16.9)	11 (16.2)	.	.	.	.
Working characteristics						
Residency with private orientation n (%)	88 (76.5)	52 (76.5)	1.003	0.994	0.495	2.034
Engaging in dual practice n (%)	88 (76.5)	45 (66.2)	1.499	0.223	0.782	2.875
*Working district*				0.022		
North n (%)	75 (65.2)	28 (43.8)	2.192	0.117	0.821	5.851
Center n (%)	29 (25.2)	27 (42.2)	0.879	0.805	0.150	2.449
South n (%)	11 (9.6)	9 (14.1)	.	.	.	.

When we examined the associations between the purchasing PHI variable and the other questions (see supplementary Table 1), we found that as would logically follow, the stronger the agreement was with statements regarding the claimed benefits of PHI (for example, that it provides greater healthcare availability and more health access for those who live in relatively remote areas) the more likely a physician was to recommend it (OR = 1.92,95%CI 1.35 to 2.80, *p*-value < 0.001; OR = 1.53, 95%CI 1.11 to 2.11, p-value = 0.008 respectively). We examined the univariate association between statements regarding the degree of satisfaction one feels at being an employee in the public sector to the recommendation of PHI. The higher the agreement level with the statement: “The burden I feel in the public sector prevents me from paying my patients the attention I would like to pay them,” the more likely a physician was to recommend PHI (OR = 1.547, 95%CI 1.14 to 2.10, *p*-value = 0.005). Conversely, the higher the level of agreement was with the statement: “My working place in the public sector is financially rewarding,” the less likely a physician was to recommend PHI (OR = 0.652, 95%CI 0.464 to 0.915, *p*-value = 0.013).

With regard to the statements about job and salary satisfaction, physician’s willingness to recommend their patients to purchase a private insurance was associated to the statement: “I think my income is lower than the income of my friends outside the health system” (OR = 1.433, 95%CI 1.041 to 1.974, p-value = 0.027). Table 3 presents the results from the most stable model produced using multivariate GEE analysis. The reference group of each covariate is marked with a star. Adjusting for gender and age, the only covariates left independently and significantly associated to the promotion of PHI were: northern and central working districts (OR = 1.425, 95%CI 1.227 to 1.655, *p*-value < 0.001; OR = 1.578, 95%CI 1.414 to 1.76, p-value < 0.001) when compared to the southern region in relation to the statement “My working place in the public sector is financially rewarding” (OR = 0.918, 95%CI 0.892 to 0.945, *p*-value < 0.001); The statements claiming that PHI creates greater availability of healthcare services and more healthcare access for those who live in relatively remote areas, were found to be independently associated to the outcome (OR = 1.650, %95CI 1.161 to 2.347, p-value = 0.005; OR = 1.660, 95%CI 1.510 to 1.821, p-value < 0.001).

**Table 3:** Association between physicians’ recommendation of purchasing or using PHI, background characteristics, and responses for survey statements. Using GEE model (*n* = 183)

	B	SE	P-value	Odds Ratio	CI low	CI High
*Working district*						
North	0.391	0.097	< 0.001	1.479	1.222	1.789
Center	0.452	0.015	< 0.001	1.572	1.525	1.62
South	.	.	.	.	.	.
*Statements regarding benefits of PHI*						
PHI allows greater access to health services	0.501	0.179	0.005	1.650	1.161	2.347
PHI allows greater access to health services in relatively remote areas	0.507	0.047	< 0.001	1.660	1.510	1.821
*Statements regarding public sector work satisfaction*						
My work place in the public sector is financially rewarding	−0.058	0.011	< 0.001	0.944	0.922	0.966

### The payment driven commitment

In the univariate analysis (supplementary Table 2), no background characteristics were found to be significantly associated with the dichotomous response for the statement: “I feel more obliged to patients who pay out of pocket for the service they receive” (Table 4). Because this question elicits perceptions regarding fee-for-service healthcare, we included only dual practice physicians in the analysis. Compared to those who disagreed with this sentence, those who agreed tended to disagree with the statement: “I find working in the public sector rewarding “(OR = 0.582, 95%CI 0.37 to 0.91, *p*-value = 0.02). From the statements describing general economic and job satisfaction, only one was found to be positively associated to the outcome: “The job I perform is more valuable and important than the job my colleagues perform “(OR = 1.491, 95%CI 1.046 to 2.125, *p*-value = 0.027).

**Table 4:** Characteristics of physicians responding to the statement: “I feel more obliged to patients who pay out of their pockets for the service they receive”

			Odds Ratio	*P*-Value	CI low	CI High
	Agree	Disagree				
	*n = 40*	*n = 137*				
*Gender*						
Males n (%)	36 (90.0)	115 (83.9)	1.722	0.346	0.557	5.326
Females n (%)	4 (10.0)	22 (16.1)	.	.	.	.
Working characteristics						
Residency with private orientation n (%)	32 (82.1)	107 (79.3)	1.169	0.702	0.478	2.994
Engaging in dual practice n (%)	26 (65.0)	107 (79.3)	0.521	0.095	0.242	1.120
*Working district*				0.388		
North n (%)	24 (61.5)	74 (55.2)	2.919	0.170	0.631	13.503
Center n (%)	13 (33.3)	42 (31.3)	2.786	0.206	0.569	13.630
South n (%)	2 (5.1)	18 (13.4)	.	.	.	.

Physicians’ obligations towards patients who pay were found to be significantly positively associated more to statements regarding the benefits of the private system, compared to any other outcome examined: “In the private system, I can provide better and more devoted care than I can in the public system” (OR = 1.679, 95%CI 1.166 to 2.418, *p*-value = 0.005); “In the private system patients trust me much more than they would in the public system “(OR = 1.995, 95%CI 1.328 to 2.877, *p*-value < 0.001).

Those who declared that they felt more obliged to patients who pay also tended to agree more with one of cardinal statements dealing with general perceptions of healthcare: “Patients are more committed to a course of treatment if they pay for it out of their own pockets “(OR = 1.536, 95%CI 1.091 to 2.296, p-value = 0.036).

Due to the small size of the sample, we decided to conduct analysis of this stage without considering the residency clusters and by performing multivariate logistic regressions. Table 5 represents the model that was most fitted and stable among those we tested. Due to a collinearity between the two last statements presented in the paragraph above, only one was entered into the regression at a time, and the best model was chosen. After adjusting for age and gender, only two statements were found to be significantly associated to the obligation one has to a patient: “The job I perform is more valuable and important than the job my colleagues perform “(OR = 1.778, 95%CI 1.005 to 3.146, *p*-value = 0.048), and “In the private system patients trust me much more than they would in the public system “(OR = 2.063, 95%CI 1.221 to 3.485, p-value = 0.007).

**Table 5:** Association between physicians’ obligation to patients who pay, background characteristics and responses for survey statements. Using multivariate logistic regression (*n* = 177)

	B	SE	*P*-value	Odds Ratio	CI Low	CI High
*Background characteristics*						
Gender [Female]	−1.982	1.144	0.083	0.138	0.015	1.296
Age [Years]	−0.015	0.035	0.669	0.985	0.92	1.055
*Statements regarding job and salary satisfaction*						
The job I perform is more valuable and important than the job my colleagues perform	0.576	0.291	0.048	1.778	1.005	3.146
*Statements regarding perspective of health*						
Patients are more committed to a course of treatment if they pay out of their own pockets for it	−0.009	0.288	0.976	0.992	0.564	1.744
In the private system, patients trust me much more than they do/would in the public system	0.724	0.268	0.007	2.063	1.221	3.485
*Statements regarding satisfaction of work in the public sector*						
I find working in the public sector rewarding	−0.273	0.338	0.419	0.761	0.392	1.476

## Discussion

In recent years, the blurring of boundaries between the public and private systems has become a significant concern to a growing number of people. According to much recent literature on the topic, PPM has led to injustice, inequality, higher out-of-pocket expenditures and inefficient distribution of resources [41]. While some physicians choose to move from the public system to the private sector, others prefer dual practice [29]. Physicians perceive the potential for dual practice to cause conflicts of interests, such as over deciding whether they prefer to see their patients within the public or private sector frameworks. While most studies in this field have focused on understanding the motives behind dual practice [19, 22, 23], as presented in section 4.2, ours has shed light on the motives leading physicians to promote private healthcare services to their patients.

We hypothesized that, by assimilating the common habits of their predominant working framework, whether private or public, physicians who have specialism loaded more to the private sector would tend to offer their patients more private services than specialists loaded to the public sector. This hypothesis was not confirmed in our study, as no significant difference was found in the proportions of physicians who would recommend PHI across the two sectors. However, financial satisfaction did seem to matter, as physicians who felt less financially rewarded in the public sector tended to agree they might offer their patients services in the private sector via PHI. According to Johannessen et al. [29], the income level of physicians might be a major factor encouraging them to enter into dual practice. Our study has found that, if physicians are discontented with their income levels, this can affect not only their decisions on whether to engage in dual practice, but also the recommendations they make to their patients on a day-to-day basis. Another factor that also might play a role in these and related decisions is the sense of being valued.

Humphrey and Russell argue that the private system provides physicians with a greater sense of being valued than the public system, leading them to gravitate more to the former [22]. Since time spent in the private system is related to the number of patients physicians are willing to see, physicians may have an interest in offering more services to their patients within the private system. Our findings were consistent with this hypothesis, as the willingness of physicians to offer more private services was found to be associated with a self-perception of being less valued in the public system. Beside self-interest, the belief that the use of private services will benefit patients might also play a role in physicians offering private services. Yet, physician’s perception regarding the possible benefits of private services could also be an attempt to justify their own activity in the private sector [42]. Physicians who considered the amount of time they have with each patient insufficient and who agreed with statements regarding the benefits patients can gain from PHI (Table 3) were more likely to recommend PHI. Does this recommendation arise solely from altruistic motives, or is it rather mediated by reward-driven perceptions one might have? This question requires future research.

The role of the private market increasingly affects the pricing, costings, availability and distribution of healthcare [43]. As an increasingly integral element of healthcare provision, market forces have enabled patients to choose their preferred services from the providers they want, as long as they have the means to pay for them [44]. At the same time, it has allowed physicians and PHI providers to set the prices for the services they provide. The infusion of money and money-driven incentives into healthcare has significantly changed physicians’ perceptions towards healthcare provision [45]. Physicians’ behavior toward and commitment to patients may be partly affected by higher income incentives for them [46].

We hypothesized that physicians who engage in dual practice and receive fee-for-service compensation would be more likely to consider healthcare a commodity than a basic right, when compared to those who work only in the public system. Physicians engaging in dual practice agreed more than those working only in the public system with the statement: “A patient who is able to afford a course of treatment deserves higher access to health services.” Agreement with that statement reflects a perception of healthcare as more of a commodity than a right, and so is consistent with our hypothesis. Our findings do not allow us to determine whether this view was one held by these individuals prior to their becoming physicians or developed since.

Given the delicate nature of physician-patient relationships, trust and obligation are major factors in their success [47]. Once trust is established, the relationship creates certain obligations and legal commitments a doctor owes to a patient. Our findings highlight the significance of financial matters in this relationship. For physicians who engage in dual practice, patients’ trust is translated into “willingness to pay.” Audiey et al. show that physicians consider patients who have agreed to pay for healthcare as those who have put their health and well-being above keeping down healthcare costs [48]. According to Audiey et al., the payment physicians receive may affect their behavior towards and attention to their patients. This finding is supported by our study. The obligation to patients who pay was not only found to be associated with trust physicians perceived, but also positively associated with the way the physician perceives his work (Table 5). The more physicians valued their jobs, the more likely they were to feel committed to patients who paid. Physicians who value their own “products” better than others’ are likely to demand higher prices and feel more committed to “consumers” who pay for their services (see Table 5).

The reasons practicing in the private sector is associated with a greater sense of being valued have yet to be fully understood. It may be related to their perception of health as commodity, as they might unconsciously associate patients’ willingness to pay for services with a greater appreciation of their work. However, it could also be related to patients’ gratitude to the physician for the perceived better treatment they receive in the private sector. This issue requires careful further study in order to more fully characterize physicians’ attitudes toward healthcare, and consideration of the implications for medical training.

Working on the frontline of healthcare systems, physicians act as coordinators between their patients and the healthcare systems. Like any other stakeholder, physicians are also affected by global commodification trends and the privatization of health systems around them [49]. They are continuously exposed to market forces and are forced to adapt to a perpetually changing working environment [50]. The growth of PPM in healthcare systems is a significant example of this changing environment. While the general messaging relating to private sector involvement in health are usually diffused from the top down, physicians’ choices and recommendations to their patients can encourage the spread of PPM. Although physicians are likely to play a key role in fostering PPM in healthcare institutions from the bottom up, questions regarding physician’s prior beliefs, daily decision making, and patterns of behavior and their effect on the growth of PPM have been scarcely studied, and this study has demonstrated the value of further research in this area.

### Study limitations

The findings of the quantitative research also provide perceptions reported from a telephone interview. The rate of response to the telephone interview was not high (53% from physicians in private sector, and 56% from physicians within public sector), which may affect the degree of its representativeness.

## Conclusions

Physicians, major key players in healthcare systems, have contributed very significantly to the spread of PPM, either directly engaging in dual practice and spending more time in the private sector, or indirectly through recommending PHI uptake to their patients. Our study has provided important insights into physician’s perceptions and decisions motivating them to recommend the purchase and use of PHI to their patients through a case study of the Israeli healthcare system. Achieving an understanding of physicians’ daily experience of PPM and its consequences, provide a platform for further studies internationally on the role physicians play in PPM processes, aiding policymakers in their efforts to regulate dual practice and strengthen public healthcare systems around the world.

## Supplementary Information

**Additional file 1 Supplementary Table 1** Summary of survey responses by recommendation for a patient to use or purchase PHI. **Supplementary Table 2** Summary of survey responses by obligation to patients who pays fee-for-service (among physicians engaged in dual practice).

## Data Availability

The datasets generated and analyzed during the current study are available from the corresponding author on reasonable request.

## Abbreviations

PPM: Private-public mix
PFI: Private finance initiative
PHI: Private health insurance
IMA: Israeli Medical Association
GEE: Generalized estimating equations
QIC: Quasilikelihood independence model criterion
ROC: Receiver operator curve
