# Supplementary material for: What motivates physicians to propose private services in a mixed private-public healthcare system? A mixed methods study
Source: BMC Health Serv Res. 2022 Jan 10;22:51. doi: 10.1186/s12913-022-07474-9 (PMC8750864; doi:10.1186/s12913-022-07474-9)
Supplement: Supplementary file 1 — Additional file 1 Supplementary Table 1 Summary of survey responses by recommendation for a patient to use or purchase PHI. Supplementary Table 2 Summary of survey responses by obligation to patients who pays fee-for-service (among physicians engaged in dual practice). [file 12913_2022_7474_MOESM1_ESM.docx]

**Supplementary Materials**

**Supplementary table 1**

Summary of survey responses by recommendation for a patient to use or purchase PHI

|  |  | Will you recommend your patient use/purchase PHI? | | Odds Ratio | p-value | Low limit | High limit |
| --- | --- | --- | --- | --- | --- | --- | --- |
|  |  | **Yes** | **No** |  |  |  |  |
|  |  | *n = 115* | *n= 68* |  |  |  |  |
| *1.Statements regarding benefits of PHI* |  |  |  |  |  |  |  |
| 1.1. PHI allows greater access to health services (Mean ± S.D) | | 3.42 ± 0.74 | 2.97 ± 0.91 | 1.920 | < 0.001 | 1.314 | 2.800 |
| 1.2. PHI allows greater access to health services in relatively remote areas (Mean ± S.D) |  | 3.00 ± 0.96 | 2.57 ± 1.04 | 1.537 | 0.008 | 1.119 | 2.111 |
| *2.Statements regarding satisfaction of work in the public sector* |  |  |  |  |  |  |  |
| 2.1. In the past few years, my work in the public sector has become busier (Mean ± S.D) |  | 3.17 ± 0.91 | 3.17 ± 0.96 | 0.997 | 0.986 | 0.721 | 1.379 |
| 2.2. The burden I feel in the public sector prevents me from paying my patients the attention I would like to (Mean ± S.D) |  | 2.88 ± 0.95 | 2.44 ± 1.07 | 1.547 | 0.005 | 1.140 | 2.100 |
| 2.3. My work in the public sector is financially rewarding (Mean ± S.D) | | 1.94 ± 0.84 | 2.29 ± 0.98 | 0.652 | 0.013 | 0.464 | 0.915 |
| 2.4. I find work in the public sector rewarding (Mean ± S.D) |  | 2.11 ± 0.78 | 2.35 ± 0.87 | 0.699 | 0.058 | 0.482 | 1.013 |
| *3.Statements regarding job and salary satisfaction* |  |  |  |  |  |  |  |
| 3.1. I feel burnout due to the burdens I have in my workplace (Mean ± S.D) | | 2.63 ± 1.02 | 2.5 ± 1.11 | 1.147 | 0.366 | 0.852 | 1.543 |
| 3.2. I think my income is lower than that of my friends outside the health system (Mean ± S.D) |  | 2.24 ± 099 | 1.89 ± 1.00 | 1.433 | 0.027 | 1.041 | 1.974 |
| 3.3. I deserve a higher salary than my current one (Mean ± S.D) |  | 3.26 ± 0.83 | 3.13 ± 0.91 | 1.191 | 0.319 | 0.845 | 1.680 |
| 3.4. The job I perform is more valuable and important than the ones my colleagues perform (Mean ± S.D) |  | 2.30 ± 1.00 | 2.37 ±1.10 | 0.937 | 0.669 | 0.695 | 1.263 |
| *4.Statements regarding perspective of health* |  |  |  |  |  |  |  |
| 4.1. A patient who can afford to pay for treatment deserves greater access (Mean ± S.D) |  | 2.52 ± 1.19 | 2.18 ± 1.19 | 1.267 | 0.071 | 0.980 | 1.638 |
| 4.2. Patients are more committed to a treatment if they pay for it out of their own pockets (Mean ± S.D) |  | 2.49 ± 1.10 | 2.40 ± 1.02 | 1.078 | 0.616 | 0.805 | 1.444 |
| 4.3. In the private system, I can provide better and more devoted care than I could in the public system (Mean ± S.D) |  | 2.20 ± 1.12 | 1.90 ± 1.12 | 1.250 | 0.136 | 0.932 | 1.676 |
| 4.4. In the private system, patients trust me much more than they would in the public system (Mean ± S.D) |  | 2.25 ± 1.11 | 2.00 ± 1.02 | 1.249 | 0.155 | 0.919 | 1.696 |
|  |  |  |  |  |  |  |  |

**Supplementary table 2**

Summary of survey responses by obligation to patients who pays fee-for-service (among physicians engaged in dual practice).

|  |  | I feel more obliged to a patient who pays out-of-pocket money for the service he/she gets | | Odds Ratio | p-value | Low limit | High limit |
| --- | --- | --- | --- | --- | --- | --- | --- |
|  |  | Agree | Disagree |  |  |  |  |
|  |  | *n = 26* | *n= 107* |  |  |  |  |
| *1.Statements regarding benefits of PHI* |  |  |  |  |  |  |  |
| 1.1. PHI allows greater access to health services (Mean ± S.D) | | 3.27 ± 0.87 | 3.25 ± 0.82 | 1.023 | 0.917 | 0.669 | 1.565 |
| 1.2. PHI allows greater access for health services in relatively remote areas (Mean ± S.D) |  | 3.02 ± 1.09 | 2.82 ± 0.99 | 1.226 | 0.299 | 0.835 | 1.800 |
| *2.Statements regarding satisfaction of work in the public sector* |  |  |  |  |  |  |  |
| 2.1. In the past few years, my work in the public sector has become busier (Mean ± S.D) |  | 3.12 ± 0.92 | 3.20 ± 0.91 | 0.911 | 0.633 | 0.622 | 1.335 |
| 2.2. The burdens I feel in the public sector prevent me from paying my patients the attention I would like to (Mean ± S.D) |  | 2.84 ± 1.02 | 2.68 ± 1.04 | 1.159 | 0.415 | 0.813 | 1.653 |
| 2.3. My work in the public sector is financially rewarding (Mean ± S.D) | | 1.94 ± 0.84 | 2.05 ± 0.92 | 0.92 | 1.001 | 0.994 | 0.677 |
| 2.4. I find work in the public sector rewarding (Mean ± S.D) |  | 1.92 ± 0.76 | 2.37 ± 0.82 | 0.582 | 0.020 | 0.370 | 0.917 |
| *3.Statements regarding job and salary satisfaction* |  |  |  |  |  |  |  |
| 3.1. I feel burnout from the burdens I have in my work (Mean ± S.D) | | 2.91 ± 0.89 | 2.46 ± 1.07 | 1.537 | 0.024 | 1.058 | 2.233 |
| 3.2. I think my income is lower than that of my friends outside the health system (Mean ± S.D) |  | 2.15 ± 0.88 | 2.04 ± 1.04 | 1.114 | 0.549 | 0.783 | 1.586 |
| 3.3. I deserve a higher salary than my current one (Mean ± S.D) |  | 3.30 ± 0.73 | 3.17 ± 0.89 | 1.199 | 0.414 | 0.776 | 1.851 |
| 3.4. The job I perform is more valuable and important than the ones my colleagues perform (Mean ± S.D) |  | 2.61 ± 0.96 | 2.19 ±1.04 | 1.491 | 0.027 | 1.046 | 2.125 |
| *4.Statements regarding perspective of health* |  |  |  |  |  |  |  |
| 4.1. A patient who can afford to pay for treatment deserves greater access (Mean ± S.D) |  | 2.75 ± 1.17 | 2.36 ± 1.19 | 1.321 | 0.073 | 0.974 | 1.793 |
| 4.2. Patients are more committed to treatment if they pay for it out of their own pockets (Mean ± S.D) |  | 2.82 ± 1.09 | 2.33 ± 1.04 | 1.559 | 0.015 | 1.091 | 2.226 |
| 4.3. In the private system, I can provide better and more devoted care than I could in the public system (Mean ± S.D) |  | 2.65 ± 1.07 | 1.92 ± 1.07 | 1.797 | <0.001 | 1.291 | 2.500 |
| 4.4. In the private system, patients trust me much more than they would in the public system (Mean ± S.D) |  | 2.74 ± 0.99 | 1.96 ± 1.06 | 1.938 | <0.001 | 1.365 | 2.752 |
|  |  |  |  |  |  |  |  |
